# Supplementary material for: Limited Added Diagnostic Value of Whole Genome Sequencing in Genetic Testing of Inherited Retinal Diseases in a Swiss Patient Cohort
Source: Int J Mol Sci. 2024 Jun 13;25(12):6540. doi: 10.3390/ijms25126540 (PMC11203445; doi:10.3390/ijms25126540)

Figure S1. Screenshots of the copy number variants analysis on JSI Sequence Pilot from exome sequencing data for the index proband of families 16, 17, 18, and 19 for genes *CNGA1*, *EYS*, *CLN3*, and *KIF11*, respectively.

Family 16

Gene: *CNGA1*

Variant: NM_001142564.1:c.507-1368_*6475del (exons 6-10)

Size: 14’834 bp

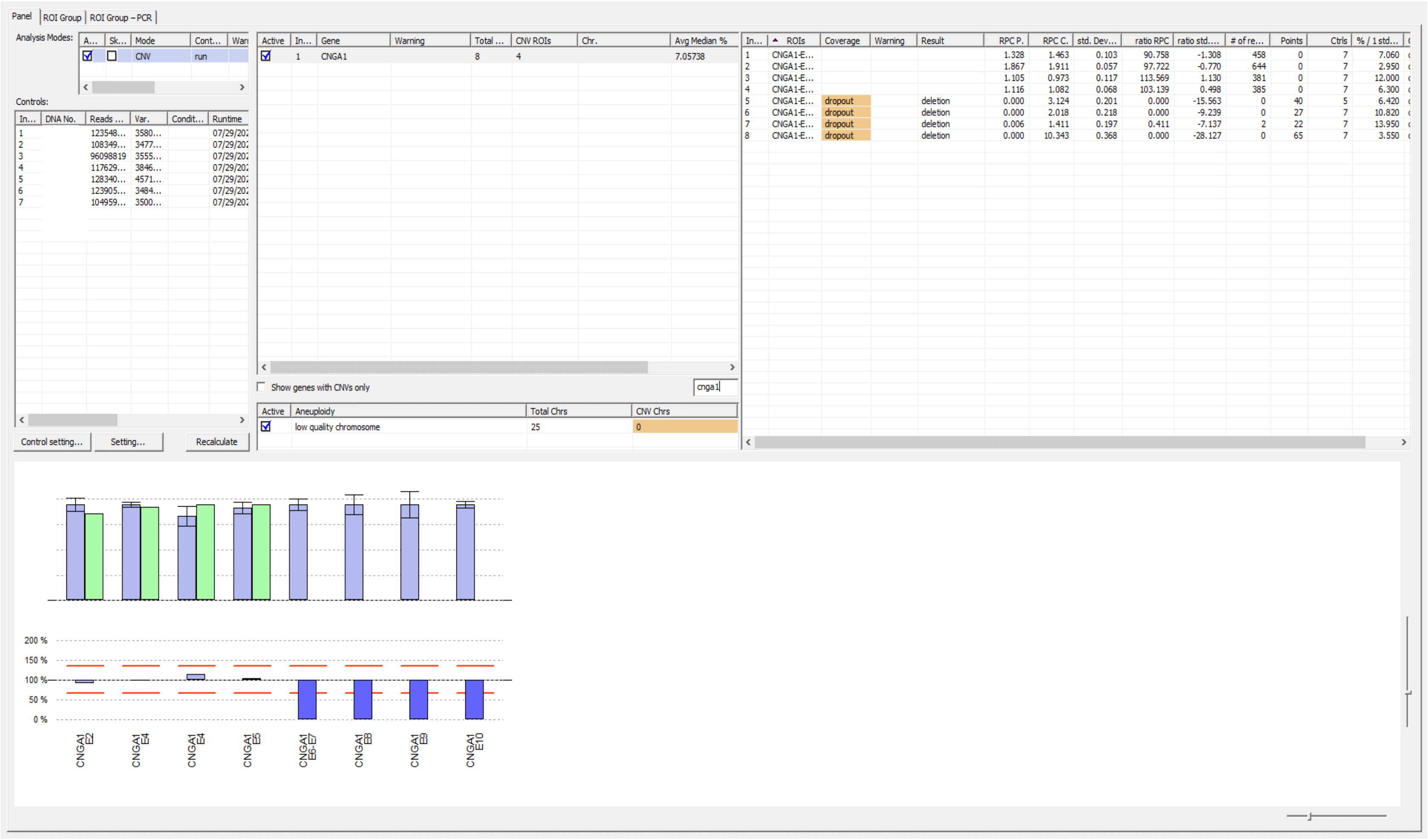

Family 17

Gene: *EYS*

Variant: NM_001142800.1:c.-203835_863-36502del (exons 1-5)

Size: 468’653 bp

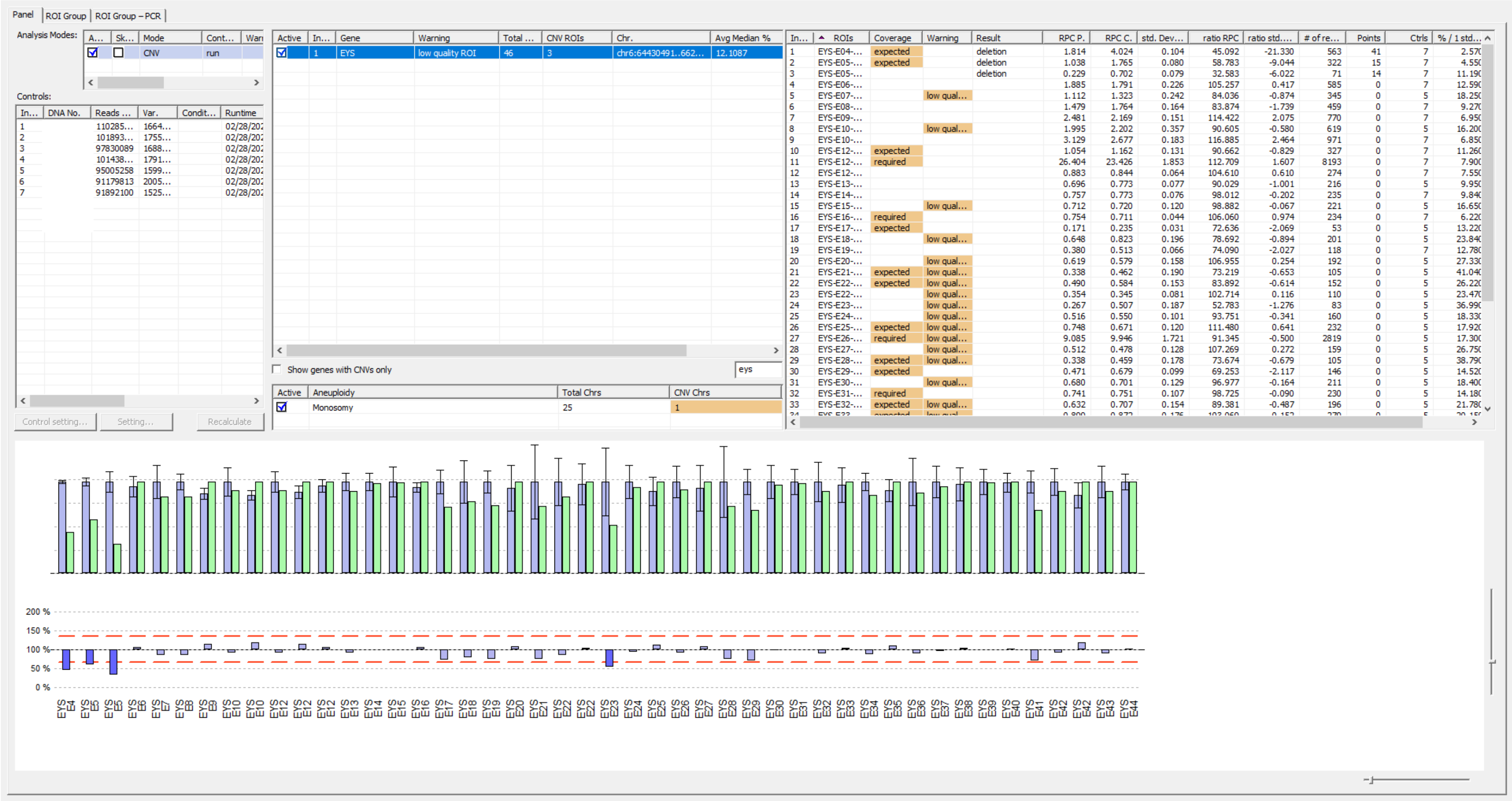

Family 18

Gene: *CLN3*

Variant: NM_001042432.1:c.461-280_677+382del (exons 8-9)

Size: 965 bp

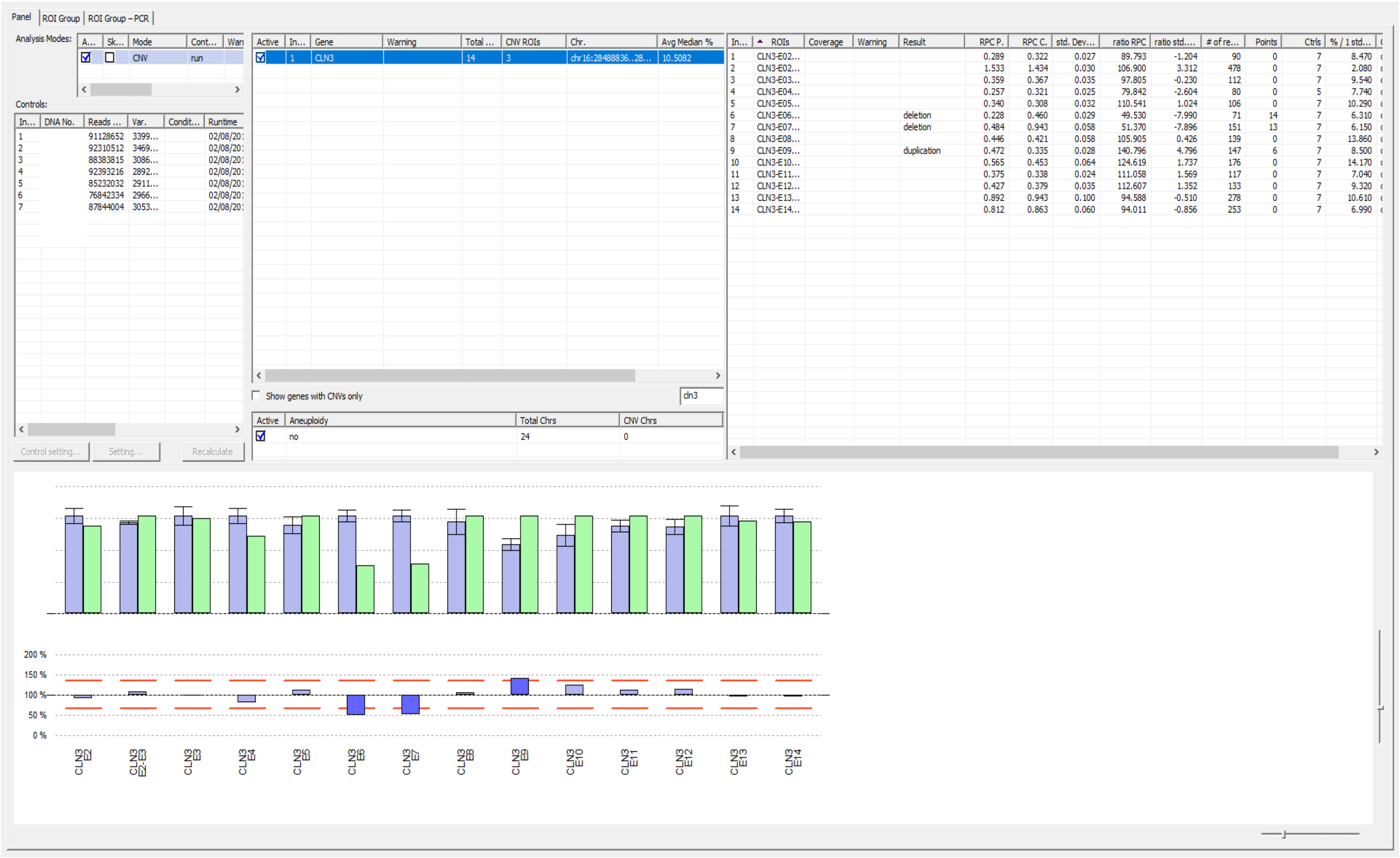

Family 19

Gene: *KIF11*

Variant: NM_004523.4:c.-5104_78-494delinsGCATGAGCCTGAGATCAAGG (exon 1)

Size: 17’499 bp

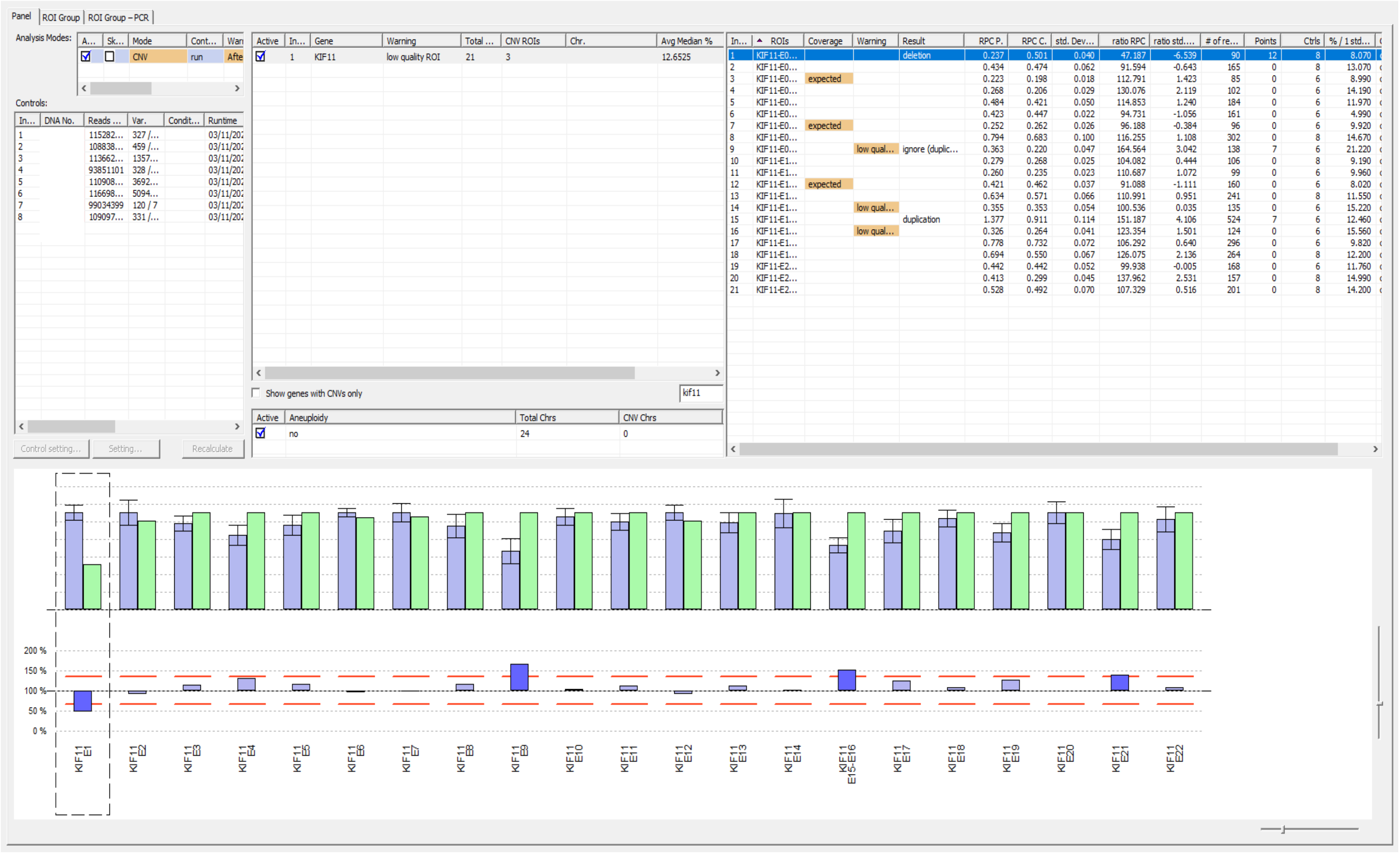

Supplement: Supplementary file 1 [file ijms-25-06540-s001.zip › Manuscript_WGS_Figure_S1.pdf]
