# Supplementary material for: Limited Added Diagnostic Value of Whole Genome Sequencing in Genetic Testing of Inherited Retinal Diseases in a Swiss Patient Cohort
Source: Int J Mol Sci. 2024 Jun 13;25(12):6540. doi: 10.3390/ijms25126540 (PMC11203445; doi:10.3390/ijms25126540)
Supplement: Supplementary file 1 [file ijms-25-06540-s001.zip › Manuscript_WGS_Table_S2.pdf]

Table S2. List of intergenic regions of interest for inherited retinal dystrophies.

chromosome	Start_hg19	End_hg19	Reported phenotype	Source
chr1	94605000	94630000	STGD	Identification and characterization of a novel retina-specific lncRNA upstream ABCA4 with a potential role in ABCA4-associated inherited retinal disease
chr1	197559000	197605000	*CRB1*-related disorders	ARVO2022 - Victor Lopez Soriano
chr5	3224781	3227534	North Carolina macular dystrophy	10.1101/2022.03.08.481329
chr5	3426820	3428709	North Carolina macular dystrophy	10.1101/2022.03.08.481329
chr5	3488122	3490861	North Carolina macular dystrophy	10.1101/2022.03.08.481329
chr5	3529321	3531958	North Carolina macular dystrophy	10.1101/2022.03.08.481329
chr5	3620584	3622714	North Carolina macular dystrophy	10.1101/2022.03.08.481329
chr5	3628499	3630933	North Carolina macular dystrophy	10.1101/2022.03.08.481329
chr5	3649808	3651837	North Carolina macular dystrophy	10.1101/2022.03.08.481329
chr5	3729781	3732646	North Carolina macular dystrophy	10.1101/2022.03.08.481329
chr5	3786502	3788892	North Carolina macular dystrophy	10.1101/2022.03.08.481329
chr5	4424696	4425672	North Carolina macular dystrophy	10.1101/2022.03.08.481329
chr6	100036084	100037115	North Carolina macular dystrophy	10.1101/2022.03.08.481329
chr6	100038442	100039290	North Carolina macular dystrophy	10.1101/2022.03.08.481329
chr6	100040717	100041240	North Carolina macular dystrophy	10.1101/2022.03.08.481329
chr6	100043728	100044622	North Carolina macular dystrophy	10.1101/2022.03.08.481329
chr6	100046527	100047100	North Carolina macular dystrophy	10.1101/2022.03.08.481329
chr6	100065704	100067743	North Carolina macular dystrophy	10.1101/2022.03.08.481329
chr6	100093481	100094893	North Carolina macular dystrophy	10.1101/2022.03.08.481329
chr6	100197194	100199999	North Carolina macular dystrophy	10.1101/2022.03.08.481329
chr11	31989150	31990050	Foveal/macular phenotype	ARVO2022 - Victor Lopez Soriano
chr17	57499214	57510765	RP17	OMIM (600852)
