# Supplementary material for: Limited Added Diagnostic Value of Whole Genome Sequencing in Genetic Testing of Inherited Retinal Diseases in a Swiss Patient Cohort
Source: Int J Mol Sci. 2024 Jun 13;25(12):6540. doi: 10.3390/ijms25126540 (PMC11203445; doi:10.3390/ijms25126540)
Supplement: Supplementary file 1 [file ijms-25-06540-s001.zip › Manuscript_WGS_Table_S3.pdf]

Table S3: Interesting findings in undiagnosed families. All variant lines represent one allele. Abbreviations: Fam., family; Clinical phen., clinical phenotype; Age at ref., age at referral; gnomAD all (%), genome aggregation database overall minor allele frequency in percentage; gnomAD max (%), genome aggregation database highest minor allele frequency in percentage; in-house (%), in-house database overall minor allele frequency in percentage; IRD (%), in-house database inherited retinal dystrophy patients-only minor allele frequency in percentage; ACMG, American College of Medical Genetics and Genomics guidelines; HGMD, Human Gene Mutation Database; PMID, publication PubMed ID; Seg., segregation; MD, macula dystrophy; CACD, central areolar choroidal dystrophy; RP, retinitis pigmentosa; EVR, exudative vitreoretinopathy; OPA, optic atrophy; CD, corneal dystrophy; VUS, variant of unknown significance; P, pathogenic; LP, likely pathogenic; A, association; ERA, established risk allele; Y, the variant segregates with disease within the family; N, the variant does not segregate with disease within the family; NA, not available. *****, variants in regions covered exclusively by genome sequencing have only been sequenced for patients affected by inherited retinal dystrophies; therefore, the overall in-house database minor allele frequency and the IRD-only minor allele frequency have the same value.

Fam.	Clinical phen.	Age at ref.	Gene	Variant	gnomAD all (%)	gnomAD max (%)	In-house (%)	IRD (%)	ACMG	HGMD	ClinVar	PMID	Seg.
20	CRD	45	*GUCY2D*	NM_000180.3:c.2506C>T	0,002	0,004	0,06	0,11	VUS	-	VUS		NA
			RD3	NM_183059.2:c.139C>T	0,966	1,987	1,52	1,21	B	-	VUS/B		
			AGBL5	NM_021831.6:c.109_730-116inv	0	0	0,06	0,11	-	-	-		
			CACNA2D4	NM_172364.4:c.3323A>C	0,018	0,037	0,13	0,11	VUS	-	VUS		
21	MD	43	*GUCY2D*	NM_000180.3:c.1081G>A	0,042	0,077	0,19	0,22	VUS	P?	VUS	32483926	N
			RD3	NM_183059.2:c.584A>T	0,953	2,811	1,33	0,99	B	P?	B/LB	24265693	NA
			PRPH2	NM_000322.4:c.-130_-128delinsGG	0	0	0,10	0,19	VUS	-	-		Y
			ABCA4	NM_000350.2:c.5603A>T	4,219	6,647	5,88	4,97	B	A	LP/ERA/ VUS/B/LB	28446513	N
22	CRD	33	*CRB1*	NM_201253.2:c.1843G>A	0	0	0,06	0,11	LP	-	LP		NA
			CRB1	NM_201253.2:c.71-15955G>A	0,057	0,118	0,76*	0,76*	VUS	-	-		
			CRB1	NM_201253.2:c.1172-23325A>C	0	0	0,76*	0,76*	VUS	-	-		
			AHI1	NM_017651.4:c.3425del	0	0	0,06	0,11	LP	-	-		
23	VMD	43	*CTNNA1*	NM_001903.4:c.1063-18480T>G	0	0	0,76*	0,76*	VUS	-	-		NA
			CTNNA1	NM_001903.4:c.2434-21C>A	0	0	0,06	0,11	VUS	-	-		
24	MD	27	*ABCA4*	NM_000350.2:c.1928T>G	0,157	0,302	0,13	0,22	LP	P	LP/VUS/LB		NA
			ABCA4	NM_000350.2:c.6147+527T>C	0,214	0,363	0,76*	0,76*	VUS	-	-		
			ABCA4	NM_000350.2:c.5603A>T	4,219	6,647	5,88	4,97	B	A	LP/ERA/ VUS/B/LB	28446513	
			CNGB3	NM_019098.4:c.418C>T	0,004	0,007	0,06	0,11	VUS	-	VUS		
25	MD	11	*PITPNM3*	NM_031220.3:c.2000G>A	0	0	0,06	0,11	VUS	-	VUS		N
			GUCA1B	NM_002098.5:c.386G>A	0,0106	0,0217	0,06	0,11	VUS	-	VUS		
26	MD	34	*ABCA4*	NM_000350.2:c.3755A>T	0,002	0,006	0,13	0,22	VUS	-	VUS		NA
			ABCA4	NM_000350.2:c.[3863-1094T>A;3863-1203G>T]	0	0	0,76*	0,76*	VUS	-	-		
			SNRNP200	NM_014014.4:c.3932C>T	0	0	0,06	0,11	VUS	-	-		

27	EVR	9	*FZD4*	NM_012193.3:c.-289G>C	0	0	0,76*	0,76*	VUS	-	-		Y
			LRP5	NM_002335.3:c.2015C>T	0,007	0,039	0,06	0,11	VUS	-	VUS		Y
28	RP	31	*PRPF31*	NM_015629.3:c.1207G>A	0,010	0,020	0,063	0,11	VUS	-	-		N
			USH2A	NM_206933.2:c.[4252-218T>C;12066+4409C>G]	0	0	0,76*	0,76*	VUS	P	-	34758253	Y
			USH2A	NM_206933.2:c.652-22287T>C	0,083	0,236	0,76*	0,76*	VUS	-	-		Y
			CDH23	NM_022124.5:c.9629T>C	0,032	0,070	0,19	0,22	VUS	-	VUS		N
			PCDH15	NM_033056.3:c.91+11716C>T	0	0	0,76*	0,76*	VUS	-	-		N
			IMPDH1	NM_000883.3:c.402+57G>A	0,188	0,344	0,19	0,22	LB	P	LB	27032803	NA
29	MD	34	*BBS7*	NM_176824.2:c.1316A>G	0,0004	0,001	0,06	0,11	VUS	-	VUS		NA
			VPS13B	NM_152564.4:c.3874G>A	0,001	0,003	0,06	0,11	VUS	-	VUS		
			TYRP1	NM_000550.2:c.540G>T	0	0	0,06	0,11	VUS	-	-		
			CACNA2D4	NM_172364.4:c.2552-1G>A	0,002	0,003	0,06	0,11	LP	P?	VUS	31964843	
30	CRD	45	*BBS1*	NM_024649.4:c.88C>A	0,002	0,005	0,06	0,11	VUS	-	VUS		NA
31	STGD	49	*PROM1*	NM_006017.2:c.2358C>T	0,045	0,512	0,13	0,22	B	-	-		NA
			PROM1	NM_006017.2:c.2358C>T	0,045	0,512	0,13	0,22	B	-	-		
32	RP	33	*TREX1*	NM_033629.4:c.581C>T	0,001	0,004	0,06	0,11	VUS	-	VUS		NA
			KCNV2	NM_133497.3:c.1381G>A	0,012	0,024	0,32	0,55	P	P	-	17896311	
33	RP	34	*KCNV2*	NM_133497.3:c.1381G>A	0,012	0,024	0,32	0,55	P	P	-	17896311	NA
34	MD	34	*ABCA4*	NM_000350.2:c.4462T>C	0,001	0,002	0,13	0,22	P	P	P/LP	9973280	NA
			GUCY2D	NM_000180.3:c.1567-9A>G	0,0004	0,001	0,06	0,11	VUS	-	LB		
35	MD	45	*ABCA4*	NM_000350.2:c.4771G>A	0,252	1,437	0,19	0,11	VUS	P?	LP/VUS/LB	28118664	NA
			BBS12	NM_152618.2:c.1859A>G	0,052	0,088	0,13	0,22	VUS	-	VUS/LB		
36	EVR	6	*LRP5*	NM_002335.3:c.3829G>T	0,005	0,010	0,06	0,11	VUS	-	VUS		Y
			FZD4	NM_012193.3:c.[97C>T;502C>T]	1,617	4,624	1,33	1,66	B	A	LB/B	28982955	Y
			VPS13B	NM_152564.4:c.10568A>G	0,017	0,031	0,06	0,11	VUS	-	VUS		NA
			COL9A3	NM_001853.3:c.412C>T	0,002	0,004	0,06	0,11	VUS	-	VUS		NA
37	RP	32	*FLVCR1*	NM_014053.3:c.1092+5G>A	0,025	0,044	0,51	0,77	P	P	P/LP	23591405	NA
			CLN3	NM_001042432.1:c.461-280_677+382del	0,125	0,556	1,56*	1,56*	P	P	-	33507216	
38	DHDD	43	*PIKFYVE*	NM_015040.3:c.4511G>A	0	0	0,06	0,11	P	P	P	35985662	NA
			MPDZ	NM_003829.4:c.1666G>A	0,001	0,002	0,06	0,11	VUS	-	-		
			MPDZ	NM_003829.4:c.3508C>T	0,001	0,005	0,06	0,11	P	-	P		
39	STGD	10	*ABCA4*	NM_000350.2:c.3322C>T	0,013	0,020	0,13	0,22	P	P	P/LP	35985662	Y
			ABCA4	NM_000350.2:c.[1555-5882C>A;1555-5784C>G]	0	0	0,76*	0,76*	VUS	-	-		Y
40	MD	33	*ADGRV1*	NM_032119.3:c.7882G>C	0	0	0,06	0,11	VUS	-	-		NA
			ADGRV1	NM_032119.3:c.23-14288T>A	0	0	0,76*	0,76*	VUS	-	-		
			LAMA1	NM_005559.3:c.415C>T	0,028	0,054	0,06	0,11	VUS	-	VUS		
41	RP	43	*MAK*	NM_001242957.2:c.174T>G	0,023	0,049	0,12	0,19	VUS	-	VUS		NA
			CDHR1	NM_033100.3:c.783G>A	0,305	0,589	0,51	0,77	LP	P	P/LP/VUS	29555955	
			IMPDH1	NM_000883.3:c.754G>T	0,009	0,042	0,06	0,11	VUS	-	VUS/LB		
42	MD	32	*TIMP3*	NM_000362.4:c.205-3117T>C	0	0	0,76*	0,76*	VUS	-	-		NA
43	MD	67	*IMPG2*	NM_016247.3:c.-131G>A	0,019	0,690	0,24	0,38	VUS	-	VUS		NA
44	RP	30	*MMACHC*	NM_015506.2:c.472T>C	0,021	0,084	0,13	0,22	LP	-	VUS		NA
			USH2A	NM_206933.2:c.4390_4391delinsCT	0	0	0,06	0,11	VUS	-	-		

			CC2D2A	NM_001080522.2:c.2162C>T	0,003	0,028	0,06	0,11	VUS	-	VUS		
			CDH23	NM_022124.5:c.[7849G>C;9629T>C]	0,004	0,055	0,06	0,11	VUS	-	VUS		
45	MD	37	*CNGB3*	NM_019098.4:c.886_896delinsT	0,005	0,014	0,06	0,11	P	-	P/LP		NA
			USH2A	NM_206933.2:c.6416C>T	0,003	0,012	0,06	0,11	VUS	-	VUS		
46	MD	46	*ABCA4*	NM_000350.2:c.317A>T	0,119	0,864	0,13	0,22	VUS	P?	VUS/B/LB	28118664	NA
			PDE6A	NM_000440.2:c.282G>A	0,029	0,048	0,06	0,11	VUS	-	VUS		
			BBS9	NM_198428.2:c.694A>G	0,002	0,014	0,06	0,11	VUS	-	-		
			CEP290	NM_025114.3:c.3322A>G	0	0	0,06	0,11	VUS	-	-		
47	WGN	30	*PDE6A*	NM_000440.2:c.487T>C	0	0	0,06	0,11	VUS	-	-		NA
			USH1G	NM_173477.2:c.436C>T	0,001	0,003	0,13	0,22	VUS	-	VUS		
			ABCA4	NM_000350.2:c.5603A>T	4,219	6,647	5,88	4,97	B	A	LP/ERA/ VUS/B/LB	28446513	
48	OCA	32	*OCA2*	NM_000275.3:c.[574-53C>G;1327G>A]	0,306	0,510	0,51	0,66	LP	P	P/LP	8302318	Y
			TYR	NM_000372.5:c.575C>A	25,02	45,07	37,99	34,33	VUS	A	P/VUS/B/ LB	35027574	Y
			TYR	NM_000372.5:c.1205G>A	17,65	27,28	20,54	18,21	VUS	A	VUS/B/LB	35027574	Y
			ABCA4	NM_000350.2:c.573C>T	0,004	0,029	0,06	0,11	VUS	-	VUS/LB		NA
49	RD	2	*CRB1*	NM_001257965.1:c.291_299del	0,062	0,111	0,19	0,22	LP	P	P/LP/LB	23379534	Y
			CRB1	NM_001257965.1:c.[-213+8068G>A;781+4086C>G]	0	0	0,76*	0,76*	VUS	-	-		Y
			ABCC6	NM_001171.5:c.2294G>A	0,004	0,006	0,06	0,11	P	P	P	11536079	NA
			ABCA4	NM_000350.2:c.5603A>T	4,219	6,647	5,88	4,97	B	A	LP/ERA/ VUS/B/LB	28446513	NA
50	EVR	1	*TMEM67*	NM_153704.5:c.622A>T	0,017	0,037	0,06	0,11	P	P	P	17397051	NA
			AIPL1	NM_014336.4:c.834G>A	0,034	0,061	0,19	0,34	P	P	P	10873396	
			ABCA4	NM_000350.2:c.5603A>T	4,219	6,647	5,88	4,97	B	A	LP/ERA/ VUS/B/LB	28446513	
51	LHON	26	*COL18A1*	NM_001379500.1:c.3664C>T	0,002	0,014	0,07	0,11	VUS	-	VUS		NA
52	USH	10	*PRPF6*	NM_012469.3:c.1069A>G	0	0	0,13	0,11	VUS	-	-		N
53	CHM	58	*IMPDH1*	NM_000883.3:c.130G>C	0	0	0,07	0,12	VUS	-	VUS		NA
54	MD	39	*CNGA3*	NM_001298.2:c.1181C>T	0	0	0,06	0,11	LP	-	-		NA
			PRDM13	NM_021620.2:c.-4096T>C	0	0	0,76*	0,76*	VUS	-	-		
55	COD	10	*PCDH15*	NM_033056.3:c.1812A>C	0,001	0,016	0,06	0,11	VUS	-	VUS		NA
			AIPL1	NM_014336.4:c.828G>C	0,001	0,010	0,06	0,11	VUS	-	VUS		
56	RP	61	*RHO*	NM_000539.3:c.959C>A	0,010	0,042	0,13	0,22	VUS	P?	LB/VUS	16123440	NA
			ABCC6	NM_001171.5:c.3515C>T	0,002	0,003	0,06	0,11	VUS	-	VUS		
			KIZ	NM_018474.4:c.907A>C	0,001	0,002	0,06	0,11	VUS	-	-		
57	MD	13	*CNGB1*	NM_001297.4:c.2153G>C	0,014	0,091	0,19	0,22	VUS	P?	VUS/LB	28127548	NA
			CNGB1	NM_001297.4:c.3419C>T	0,001	0,011	0,06	0,11	VUS	-	VUS		
			MPDZ	NM_003829.4:c.5683A>C	0,006	0,023	0,06	0,11	VUS	-	VUS		
			MPDZ	NM_003829.4:c.5959G>A	0,001	0,017	0,06	0,11	VUS	-	VUS		
			EYS	NM_001142800.1:c.6560C>G	0	0	0,06	0,11	VUS	-	-		
58	MD	55	*ABCA4*	NM_000350.2:c.3755A>T	0,002	0,006	0,13	0,22	VUS	-	VUS		NA
			ABCA4	NM_000350.2:c.3863-1094T>A	0	0	1,52	1,52	VUS	-	-		
			RAX2	NM_032753.3:c.76A>C	0,005	0,016	0,06	0,11	VUS	-	VUS/LB		

59	RP	23	*PDE6B*	NM_000283.3:c.1118T>A	0,001	0,001	0,06	0,11	VUS	-	-		NA
60	RP	20	*OPA1*	NM_015560.2:c.1146A>G	0,059	0,076	0,06	0,11	P	P	P/LP/VUS	17722006	NA
			EXOSC2	NM_014285.6:c.454G>A	0,001	0,011	0,06	0,11	VUS	-	-		
